# Low frequency of mutation of epidermal growth factor receptor (*EGFR*) and arrangement of anaplastic lymphoma kinase (*ALK*) in primary pulmonary lymphoepithelioma‐like carcinoma

**DOI:** 10.1111/1759-7714.13271

**Published:** 2019-12-03

**Authors:** Kai Yin, Hui‐Bo Feng, Lin‐Lin Li, Yu Chen, Zhi Xie, Zhi‐Yi Lv, Wei‐Bang Guo, Dan‐Xia Lu, Xue‐Ning Yang, Wen‐Qing Yan, Yi‐Long Wu, Xu‐Chao Zhang

**Affiliations:** ^1^ The Second School of Clinical Medicine Southern Medical University Guangzhou China; ^2^ Guangdong Lung Cancer Institute, Guangdong Provincial Key Laboratory of Translational Medicine in Lung Cancer, Medical Research Center, Cancer Center Guangdong Provincial People's Hospital & Guangdong Academy of Medical Sciences Guangzhou China

**Keywords:** Anaplastic lymphoma kinase (*ALK*), epidermal growth factor receptor (*EGFR*), pulmonary lymphoepithelioma‐like carcinoma (PLELC)

## Abstract

**Background:**

Primary pulmonary lymphoepithelioma‐like carcinoma (PLELC) is a rare and unique subtype of lung cancer. However, the prevalence of driver alterations, such as epidermal growth factor receptor (*EGFR*) mutations and anaplastic lymphoma kinase (*ALK*) rearrangements, and the response of tyrosine kinase inhibitor (TKIs) in PLELC has not been thoroughly investigated.

**Method:**

We retrospectively reviewed the genetic profiles and treatment course of 330 PLELC patients at the Guangdong Lung Cancer Institute (GLCI) from 1st January, 2008 to 30th December, 2018. We searched and analyzed related literature published in PubMed and Web of Science from 1st January, 2000 and 31th August, 2019 based on their mention of “driver mutations” and “the response of TKIs to mutant PLELC”.

**Results:**

Genetic alterations of *EGFR/ALK* were tested in 203 patients (203/330, 61.5%). Five patients (5/175, 2.9%) had *EGFR* mutation and three patients (3/140, 2.1%) had *ALK* alteration. From the total of 15 articles identified from electronic searches, 1071 PLELC cases mentioned the driver mutations. *EGFR* mutation and *ALK* rearrangement were detected in 15 patients and one patient, respectively. In total, there were four *EGFR*/*ALK* mutant PLELC patients who received targeted therapy as palliative treatment at the GLCI and in the literature. However, there was disease progression in all cases one month after use of TKIs.

**Conclusion:**

The mutation rates of *EGFR* and *ALK* were low in PLELC. EGFR and ALK TKIs showed limited response in *EGFR/ALK* mutant PLELC. Further studies are needed to explore other molecular targets to optimize the therapeutic strategy for PLELC.

## Key points

- The mutation rates of *EGFR* and *ALK* in PLELC were lower than other common types of non‐small cell lung cancer.
- EGFR/ALK TKIs showed a limited response in *EGFR/ALK* mutant PLELC.
- This large cohort study demonstrates the prevalence of *EGFR* mutation and *ALK* alteration, and summarizes the response of targeted therapy in mutant PLELC patients.

## Introduction

Lymphoepithelioma‐like carcinoma (LELC) is an undifferentiated carcinoma of malignant epithelial cells which is more prevalent in the nasopharynx, though few cases arise from foregut‐derived organs such as the salivary glands, stomach, lung and thymus etc.^1^, ^2^, ^3^ Primary pulmonary lymphoepithelioma‐like carcinoma (PLELC) is a unique and rare subtype of non‐small cell lung cancer (NSCLC).^4^ It was removed from large cell lung cancer and reclassified as “other and unclassified carcinoma” in the 2015 World Health Organization classification for lung carcinoma.^5^ It was first described in 1987 and reported to be related to Epstein‐Barr virus (EBV) infection.^1^, ^6^, ^7^ Since then, around 1200 cases have been reported in the literature, mostly from Asian countries.^8^, ^9^, ^10^ Due to the rarity of this tumor, the standard treatment strategy for advanced PLELC patients remains controversial and a multidisciplinary management has been recommended.^1^, ^8^, ^11^, ^12^

Epidermal growth factor receptor (*EGFR)* and anaplastic lymphoma kinase *(ALK)* are reported to play an important role in the pathogenesis of lung cancer.^13^, ^14^, ^15^, ^16^ EGFR tyrosine kinase inhibitor (TKI) and ALK TKI have some clinical significance in the management of lung cancer.^17^, ^18^ The mutation rates of *EGFR* and *ALK* in NSCLC were reported to be 30%–40% and 6%–8%, respectively.^19^ NSCLC patients with *EGFR* mutation or *ALK* rearrangement are sensitive to EGFR TKI (such as Gefitinib, Erlotinib, Afatinib and Osimertinib) or ALK TKI (such as Crizotinib, Alectinib and Lorlatinib).^20^, ^21^, ^22^, ^23^, ^24^, ^25^, ^26^ With the development of precision medicine, targeted therapies play an increasingly important role in the treatment of advanced NSCLC.^13^, ^17^ TKIs such as EGFR TKI and ALK TKI have been approved for the first‐line treatment of advanced mutant nonsquamous NSCLC.^27^ Genetic tests for *EGFR* mutations and *ALK* rearrangements are routine for lung adenocarcinoma in clinical practice.^27^ However, only a few small scale retrospective studies have investigated the mutation rates of *EGFR* and *ALK* in PLELC. Wang *et al*.^28^ observed that no patient had *ALK* arrangement and only one patient harbored *EGFR 21 L858R* mutation among the 42 PLELC patients. Liu and colleagues^29^ reported that none of the 32 PLELC patients had mutations in *EGFR* exon 19 and exon 21. Chang *et al*.^30^ found that 17.4% (8/46) of PLELC patients harbored *EGFR* mutations, but the majority of them (7/8) were not classical *EGFR* mutation. The response of TKI in PLELC patient was seldom reported. These observations suggest that the *EGFR* and *ALK* alterations and targeted therapy in PLELC have not been thoroughly explored. A comprehensive review of the prevalence of driver mutations is essential for the management of PLELC.^31^ Consequently, we performed this study in order to investigate the prevalence of *EGFR* mutation and *ALK* alteration, and summarize the response of targeted therapy in mutant PLELC patients.

## Methods

### Patients

We retrospectively reviewed the profiles of patients who were diagnosed with PLELC at the Guangdong Lung Cancer institute (GLCI) from 1st January, 2008 to 30th December, 2018. A total of 330 primary PLELC patients were enrolled. Primary PLELC was diagnosed based on the criteria set by the World Health Organization.^5^ Nasopharyngoscopy or radiological imaging of the nasopharynx was conducted to rule out metastatic LELC from nasopharyngeal carcinoma. Pathologic tumor stage was defined according to the eighth edition of the American Joint Committee on Cancer staging system.^32^ This study was approved by the Hospital's Research Ethics Committee. All the patients provided written informed consent. We reviewed the medical records of the 330 PLELC patients and analyzed their clinicopathological information including age at diagnosis, gender, stage, status of *EGFR* mutation and *ALK* alteration, expression status of Epstein‐Barr virus‐encoding small RNA (EBERs), P63 and cytokeratins 5/6 (CK5/6).

To investigate the prevalence of driver mutations in PLELC patients, we searched the literature published in PubMed and Web of Science from 1st January, 2000 to 31th August, 2019 using a combination of the three keywords: “pulmonary lymphoepithelioma‐like,” “carcinoma” and “mutation.” The previous literature which reported the driver mutations in PLELC were analyzed. The response of targeted therapy in PLELC patients who harbored driver mutations both at the GLCI and in the literature was also analyzed. Progression‐free survival (PFS) was defined as the time from the start of the targeted therapy to disease progression or death from cancer, whichever came first.

### Immunohistochemistry staining and in situ hybridization (ISH) of EBERs

EBERs were detected using the EBV Probe in Situ Hybridization Kit (ZSBIO, Beijing). Most tumors were evaluated with immunohistochemical staining using CK5/6 (D5/16 B4, dilution: 1:400, Gene Technology, China), P63 (4A4, dilution: 1:1000, Gene Technology, China). All the tests were carried out and evaluated according to the manufacturer's instructions.

### Genetic analysis

Genetic analysis of the tumor was performed in 203 patients (203/330, 61.5%). *EGFR* mutations were detected by Sanger sequencing or amplification refractory mutation system (ARMS) (AmoyDx, Xiamen, China), as previously described.^33^ *ALK* alterations were detected by fluorescence in situ hybridization with *ALK* break apart probes and/or immunohistochemistry (IHC) staining with Ventana anti‐ALK antibody as previously described.^34^

## Results

### Clinicopathological characteristics of 330 PLELC patients

The clinicopathological characteristics of 330 patients with PLELC are presented in Table 1. There were 148 males (148/330, 44.8%) and 182 females (182/330, 55.2%). The median age at diagnosis was 53 years (range, 13–78 years). A total of 70% of PLELC patients were younger than 60 years. The 330 patients at diagnosis belonged to the following stages: 62 patients (62/330, 18.8%) were stage I, 48 patients (48/330, 14.5%) were stage II, 99 patients (99/330, 30.0%) were stage III, and 121 patients (121/330, 36.7%) were stage IV. Among the 203 patients who received a genetic test, five patients (5/175, 2.9%) were positive with *EGFR* mutation and three patients (3/140, 2.1%) were positive with *ALK* alteration. Most tumors were positive for the expression of EBERs (319/320, 99.7%) (10 patients did not undergo EBERs test), CK5/6 (218/228, 95.6%) and P63 (196/201, 97.5%).

**Table 1:** The clinicopathological characteristics of 330 primary pulmonary lymphoepithelioma‐like carcinoma (PLELC) patients

Variables	No. of patients	Percent (%)
Gender		
Male	148	44.8%
Female	182	55.2%
Age (year)		
<60	231	70.0%
≥60	99	30.0%
Stage		
I	62	18.8%
II	48	14.5%
III	99	30.0%
IV	121	36.7%
Mutation		
*EGFR*	5/175	2.9%
*ALK*	3/140	2.1%
EBERs	320	
Negative	1	0.3%
Positive	319	99.7%
CK5/6	228	
Negative	10	4.4%
Positive	218	95.6%
P63	201	
Negative	5	2.5%
Positive	196	97.5%

CK5/6, Cytokeratins 5/6; EBERs, Epstein‐Barr virus‐encoding small RNA; P63, tumor protein 63.

### *EGFR* and *ALK* alterations in PLELC patients of GLCI

As shown in Table 2, eight patients were positive with *EGFR* or *ALK* alterations at the GLCI between 1 January 2008 and 30 December 2018. There were two patients with *EGFR Exon 21 L858R* mutation (by Sanger sequencing), two patients with *EGFR Exon 20* insertion (by ARMS‐PCR) and one patient with *EGFR Exon 19* and *Exon 21* co‐mutation (by ARMS‐PCR). As for the three *ALK* positive patients, they were all detected by Ventana IHC. Among these patients, half were at stage II or III and received surgical treatment. Four patients were diagnosed at stage IV and two received targeted therapy after the genetic test.

**Table 2:** Molecular mutations in primary pulmonary lymphoepithelioma‐like carcinoma (PLELC) at the Guangdong Lung Cancer Institute (GLCI)

Patients	Mutation status	Detection method	TNM stage	EBERs
1	*EGFR 21 L858R*	Sanger sequencing	III	3+
2	*EGFR 21 L858R*	Sanger sequencing	IV	3+
3	*EGFR 20* insertion	ARMS‐PCR	IV	2+
4	*EGFR 20* insertion	ARMS‐PCR	II	3+
5	*EGFR exon 19/21*	ARMS‐PCR	III	1+
6	ALK fusion	Ventana IHC	IV	3+
7	ALK fusion	Ventana IHC	IV	3+
8	ALK fusion	Ventana IHC	II	3+

ARMS, amplification refractory mutation system; EBERs, Epstein‐Barr virus‐encoding small RNA.

### Molecular alterations of PLELC in the literature

Using the keywords of “pulmonary lymphoepithelioma‐like carcinoma” and “mutation,” we searched for literature published in PubMed and Web of Science between 1 January 2000 and 31 August 2019. A total of 15 articles which referred to driver mutations in PLELC were identified by electronic searches (Table 3). There were 1071 PLELC cases that mentioned driver mutations. Most of the patients were from Guangzhou and Hong Kong. Among the PLELC patients, *EGFR* mutation and *ALK* alteration were positive in 15 and one patient, respectively. *TP53* gene mutation was found in 21 patients (only mentioned in two articles, 21/118[17.8%]). Only one PLELC patient was positive for *KRAS* mutation. Except that, Chang *et al*.^30^ reported no alterations of *ALK*, *KRAS*, *ROS1*, and *BRAF* in 66 PLELC patients.

**Table 3:** Literature related to molecular mutations in primary pulmonary lymphoepithelioma‐like carcinoma (PLELC) published between 1 January 2000 and 31 August 2019

Authors	Area	Total cases	*EGFR*	*ALK*	*KRAS*	Other
Tam *et al*.^35^	Hong Kong, China	11	1	NA	NA	NA
Wong *et al*.^36^	Hong Kong, China	11	NA	0	NA	NA
Liang *et al*.^8^	Guangzhou, China	52	0/11	NA	NA	NA
Liu *et al*. 29	Guangzhou, China	32	0	NA	NA	NA
Wang *et al*. 28	Guangzhou, China	42	1	0	NA	NA
Fang *et al*. 37	Guangzhou, China	113	2	0	NA	NA
Chang *et al*. 30	Tai Wan, China	66	8	0	0	0 (*BRAF*)
Ose *et al*. 38	Osaka, Japan	1	0	1	NA	NA
Hong *et al*. 31	Guangzhou, China	91	0	0	NA	14 *(P53*)
Lin *et al*. 39	Guangzhou, China	39	0/19	NA	NA	NA
Lin *et al*. 40	Fuzhou, China	20	0/3	0/2	0/1	NA
Xie *et al*. 10	Guangzhou, China	429	2	0	0	NA
Zhou *et al*. 41	Guangzhou, China	27	0	0	1	7 (*P53*)
Yin *et al*. 42	Guangzhou, China	52	0	0	NA	NA
Qin *et al*.^2^	Guangzhou, China	85	1	NA	NA	NA
Total		1071	15	1	1	NA

NA, not available.

### Targeted therapy in PLELC patients with driver gene mutant

The response of TKIs in PLELC patients with driver gene mutations are summarized in Table 4. There were only four patients who received targeted therapy as a palliative treatment. Wang *et al*.^28^ reported the first case of *EGFR* mutant PLELC who had received Gefitinib, but in whom there waw no objective response was observed and progression free survival (PFS) was only one month. In the study of Quin, *et al*.^2^ there was only one patient with *EGFR* mutation who had been treated with EGFR‐TKI for just one month because of a quick disease progression. At our institution, there was one patient who received Erlotinib after she was detected positive with an *EGFR EXON* 19 deletion, but the PFS was only one month (no objective response was observed). Another *ALK* positive patient had disease progression one month after she received Crizotinib.

**Table 4:** Summary of target therapy in primary pulmonary lymphoepithelioma‐like carcinoma (PLELC) patients at the Guangdong Lung Cancer Institute (GLCI) and in the literature

Patients	Case sources	Mutation status	Target therapy	PFS (m)
1	Wang *et al*.^28^	*EGFR 21 L858R*	Gefitinib	1.0
2	Qin *et al*.^2^	*EGFR* mutation	EGFR TKI	1.0
3	GLCI	*EGFR 19 del*	Erlotinib	1.0
4	GLCI	ALK fusion	Crizotinib	1.1

PFS, progression‐free survival.

## Discussion

PLELC is a rare and unique subtype of lung cancer which is associated with EBV infections and has often been identified in young non‐smokers. Since its first report in 1987, there have been around 1200 cases documented in the literature. Due to its rarity, the prevalence of driver mutation and the response of TKIs in advanced mutant patients with PLELC has not been thoroughly investigated. To the best of our knowledge, this study is the first to summarize the prevalence of *EGFR* and *ALK* alteration and the response of TKIs in PLELC. We retrospectively analyzed the gene profiles and treatment courses of 330 PLELC patients at the GLCI. The mutation rates of *EGFR* and *ALK* were 2.9% and 2.1%, respectively. Moreover, *EGFR* mutation and *ALK* alteration were detected positive in 15 and one patient in the literature, respectively. There were four PLELC patients with driver gene mutations who received TKIs but they experienced disease progression only one month later. Altogether, these data suggest that the mutation rates of *EGFR* and *ALK* in PLELC are lower than other common types of NSCLC. TKIs showed a limited response in the PLELC patients with driver gene mutations.

In this study, we found that *EGFR* and *ALK* alteration were very rare in PLELC patients. The mutation rates of *EGFR* and *ALK* were 2.9% and 2.1% in the 330 PLELC patients of the GLCI cohort. In other studies published in the literature, *EGFR* mutation and *ALK* alteration in PLELC patients were reported positive in only 15 and one patient, respectively. The *EGFR* mutation rate in the literature ranges from 0% to 9.1% (average rate is 1.5% [15/972]). *ALK* alteration was detected positive in three PLELC patients in our study, while only one was reported in the literature by Ose *et al*.^38^ The variant rates may be due to the small sample size of each study which warrants further investigation. In the study by Gou and Wu which demonstrated the frequencies of driver mutations in non‐small cell lung cancer in China,^19^ the mutation rates of *EGFR* were 48.4% (675/1795), 4.3% (9/208), and 28.2% (570/2021) in lung adenocarcinoma, squamous cell cancer (SCC) and NSCLC, respectively. *ALK* arrangement was positive in 6.7% (114/1700), 2.0% (8/396), and 5.6% (108/1913) in lung adenocarcinoma, SCC and NSCLC, respectively. Among the 506 NSCLC patients who received *EGFR* mutation analysis in the study by Wu *et al*. the *EGFR* mutation rate was 30.04%.^43^ An *et al*. reported that the mutation rate of driver genes might differ among different histology subtypes and smoking status.^44^ *EGFR* and *ALK* alteration is rare in PLELC. The reason for this could be because PLELC might harbor a unique genetic profile which may impact on its response to targeted therapy.

In contrast to the excellent activity of TKIs in mutant lung adenocarcinoma, TKIs showed a limited response in the PLELC patients with *EGFR/ALK* mutation in this study. We analyzed the PFS of *EGFR/ALK* mutant PLELC patients who received TKIs at the GLCI and in the literature. Four patients received TKIs after they were detected positive with *EGFR/ALK* mutation. However, they experienced disease progression only one month later. According to the result of IPASS, OPTIMAL, Lux‐lung 3, and 6, the median PFS of EGFR TKI (Gefitinib, Erlotinib, and Afatinib, Dacomitinib and Osimertinib) to the *EGFR* mutant NSCLC patients was nearly 10–13 months.^20^, ^21^, ^22^, ^23^ As for *ALK* arrangement patients, the results of PROFILE 1014, PROFILE 1029 and ALEX study suggest that Crizotinib and Alectinib were sensitive for *ALK* positive NSCLC patients, and the median PFS was 11–34 months.^24^, ^25^ Based on these observations, TKIs may not be suitable for this type of lung cancer. Recently, Hong *et al*. 31 reported the genomic landscape of 91 PLELC patients by whole‐exome sequencing and targeted deep sequencing. They found that the frequent loss of type I interferon genes and mutated genes *TP53*, *NOTCH1*, and *MGA* were prevalent in PLELC. Critical pathways including NF‐κB, JAK/STAT, and cell cycle were also altered. These genomic features might be responsible for the poor efficacy of TKIs treatment in PLELC as *TP53* mutation has been reported to compromise TKI efficacy.^45^ Similar to other studies, PLELC was usually found in younger patients (age < 60 years old). The incidence rate was not significantly different between male and female patients. EBERs was positive in 99.7% (319/320) of the PLELC patients. These results support the idea that PLELC is closely associated with EBV infection. CK5/6 and P63, which are usually coexpressed in the lung SCC, were positive with 95.6% and 97.5% in PLELC patients, respectively. It is reported that PLELC might be misdiagnosed as lung SCC because they share similar immunohistochemical markers such as CK5/6 and P63.^46^ Therefore, EBERs in situ hybridization is vital for the diagnosis of PLELC and poorly differentiated lung SCC.

Our study has a few limitations. First, not all the PLELC patients received comprehensive genetic testing for all cancer related genes. We summarized the mutation rates of *EGFR/ALK* in the literature to consolidate our findings. Second, a few studies were not included because they were published in the Chinese journal and were not included in PubMed or Web of Science. According to our search results on Chinese academic websites, there were only two related studies which involved 10 *EGFR/ALK* wild‐type PLELC patients. Therefore, this would not have affected the results of our study.

In conclusion, by reviewing the genomic profiles and clinical treatment courses of PLELC patients at our institution and in the extant literature, we found that the mutation rates of *EGFR* and *ALK* were lower than other common types of NSCLC, and *EGFR/ALK* TKIs only showed a limited response in PLELC patients with *EGFR/ALK* mutation. Further efforts are warranted to explore other molecular targets to optimize the therapeutic strategy for PLELC.

## Disclosure

The authors declare that they have no competing interests.

## References

[1] Ho JC , Wong MP , Lam WK . Lymphoepithelioma‐like carcinoma of the lung. Respirology 2010; 11: 539–45. 10.1111/j.1440-1843.2006.00910.x 16916325

[2] Qin Y , Gao G , Xie X *et al* Clinical features and prognosis of pulmonary lymphoepithelioma‐like carcinoma: Summary of eighty‐five cases. Clin Lung Cancer 2019; 20: e329–37. 30683631 10.1016/j.cllc.2018.12.014

[3] Ngan RKC , Yip TTC , Wai‐Wai C *et al* Circulating Epstein‐Barr virus DNA in serum of patients with lymphoepithelioma‐like carcinoma of the lung: A potential surrogate marker for monitoring disease. Clin Cancer Res 2002; 8: 986–94. 11948104

[4] Chang YL , Wu CT , Shih JY , Lee YC . Unique p53 and epidermal growth factor receptor gene mutation status in 46 pulmonary lymphoepithelioma‐like carcinomas. Cancer Sci 2011; 102: 282–7. 21070477 10.1111/j.1349-7006.2010.01768.x

[5] Travis WD , Brambilla E , Nicholson AG *et al* The 2015 World Health Organization classification of lung tumors: Impact of genetic, clinical and radiologic advances since the 2004 classification. J Thorac Oncol 2015; 10: 1243–60. 26291008 10.1097/JTO.0000000000000630

[6] Hayashi T , Haba R , Tanizawa J *et al* Cytopathologic features and differential diagnostic considerations of primary lymphoepithelioma‐like carcinoma of the lung. Diagn Cytopathol 2012; 40: 820–5. 21433005 10.1002/dc.21670

[7] Begin LR , Eskandari J , Joncas J , Panasci L . Epstein‐Barr virus related lymphoepithelioma‐like carcinoma of lung. J Surg Oncol 1987; 36: 280–3. 2826922 10.1002/jso.2930360413

[8] Ying L , Liang W , Yujia Z *et al* Primary pulmonary lymphoepithelioma‐like carcinoma: Fifty‐two patients with long‐term follow‐up. Cancer 2015; 118: 4748–58. 10.1002/cncr.27452 22359203

[9] Kim C , Rajan A , Debrito PA , Giaccone G . Metastatic lymphoepithelioma‐like carcinoma of the lung treated with nivolumab: A case report and focused review of literature. Transl Lung Cancer Res 2016; 5: 720. 28149767 10.21037/tlcr.2016.11.06 PMC5233874

[10] Xie M , Wu X , Wang F *et al* Clinical significance of plasma Epstein‐Barr virus DNA in pulmonary lymphoepithelioma‐like carcinoma (LELC) patients. J Thorac Oncol 2018; 13: 218–27. 29191777 10.1016/j.jtho.2017.10.031

[11] Lin CY , Chen YJ , Hsieh MH , Wang CW , Fang YF . Advanced primary pulmonary lymphoepithelioma‐like carcinoma: Clinical manifestations, treatment, and outcome. J Thorac Dis 2017; 9: 123–8. 28203414 10.21037/jtd.2017.01.25 PMC5303072

[12] Chan ATC , Teo PML , Lam KC *et al* Multimodality treatment of primary lymphoepithelioma‐like carcinoma of the lung. Cancer 2015; 83: 925–9. 10.1002/(sici)1097-0142(19980901)83:5<925::aid-cncr18>3.0.co;2-x 9731896

[13] Tan WL , Jain A , Takano A *et al* Novel therapeutic targets on the horizon for lung cancer. Lancet Oncol 2016; 17: e347–62. 27511159 10.1016/S1470-2045(16)30123-1

[14] Liu SY , Dong ZY , Wu SP *et al* Clinical relevance of PD‐L1 expression and CD8+ T cells infiltration in patients with EGFR‐mutated and ALK‐rearranged lung cancer. Lung Cancer 2018; 125: 86–92. 30429043 10.1016/j.lungcan.2018.09.010

[15] Su S , Dong ZY , Xie Z *et al* Strong programmed death ligand 1 expression predicts poor response and de novo resistance to EGFR tyrosine kinase inhibitors among NSCLC patients with EGFR mutation. J Thorac Oncol 2018; 13: 1668–75. 30056164 10.1016/j.jtho.2018.07.016

[16] Dong ZY , Zhang JT , Liu SY *et al* EGFR mutation correlates with uninflamed phenotype and weak immunogenicity, causing impaired response to PD‐1 blockade in non‐small cell lung cancer. Oncoimmunology 2017; 6: e1356145. 29147605 10.1080/2162402X.2017.1356145 PMC5674946

[17] Hirsch FR , Scagliotti GV , Mulshine JL *et al* Lung cancer: Current therapies and new targeted treatments. Lancet 2017; 389: 299–311. 27574741 10.1016/S0140-6736(16)30958-8

[18] Ke EE , Wu YL . EGFR as a pharmacological target in EGFR ‐mutant non‐small‐cell lung cancer: Where do we stand now? Trends Pharmacol Sci 2016; 37: 887–903. 27717507 10.1016/j.tips.2016.09.003

[19] Gou LY , Wu YL . Prevalence of driver mutations in non‐small‐cell lung cancers in the People's Republic of China. Lung Cancer 2014; 5: 1–9. 28210137 10.2147/LCTT.S40817 PMC5217505

[20] Mok TS , Wu YL , Thongprasert S *et al* Gefitinib or carboplatin‐paclitaxel in pulmonary adenocarcinoma. N Engl J Med 2009; 361: 947–57. 19692680 10.1056/NEJMoa0810699

[21] Sun H , Wu YL . Dacomitinib in non‐small‐cell lung cancer: A comprehensive review for clinical application. Future Oncol 2019; 15: 2769–77. 31401844 10.2217/fon-2018-0535

[22] Yang JC , Sequist LV , Geater SL *et al* Clinical activity of afatinib in patients with advanced non‐small‐cell lung cancer harbouring uncommon EGFR mutations: A combined post‐hoc analysis of LUX‐Lung 2, LUX‐Lung 3, and LUX‐Lung 6. Lancet Oncol 2015; 16: 830–8. 26051236 10.1016/S1470-2045(15)00026-1

[23] Zhou C , Wu YL , Chen G *et al* Erlotinib versus chemotherapy as first‐line treatment for patients with advanced EGFR mutation‐positive non‐small‐cell lung cancer (OPTIMAL, CTONG‐0802): A multicentre, open‐label, randomised, phase 3 study. Lancet Oncol 2011; 12: 735–42. 21783417 10.1016/S1470-2045(11)70184-X

[24] Wu YL , Lu S , Lu Y *et al* Results of PROFILE 1029, a phase III comparison of first‐line crizotinib versus chemotherapy in East Asian patients with ALK‐positive advanced non‐small cell lung cancer. J Thorac Oncol 2018; 13: 1539–48. 29966800 10.1016/j.jtho.2018.06.012

[25] Solomon BJ , Mok T , Kim DW *et al* First‐line crizotinib versus chemotherapy in ALK‐positive lung cancer. N Engl J Med 2014; 371: 2167–77. 25470694 10.1056/NEJMoa1408440

[26] Peters S , Camidge DR , Shaw AT *et al* Alectinib versus crizotinib in untreated ALK‐positive non‐small‐cell lung cancer. N Engl J Med 2017; 377: 829–38. 28586279 10.1056/NEJMoa1704795

[27] National Comprehensive Cancer Network . NCCN clinical practice guidelines in oncology. (NCCN Guidelines®) Non‐Small Cell Lung Cancer). Version 1.2018. Available from URL: https://www.nccn.org/patients/guidelines/lung-metastatic/index.html.

[28] Wang L , Lin Y , Cai Q *et al* Detection of rearrangement of anaplastic lymphoma kinase (ALK) and mutation of epidermal growth factor receptor (EGFR) in primary pulmonary lymphoepithelioma‐like carcinoma. J Thorac Dis 2015; 7: 1556–62. 26543602 10.3978/j.issn.2072-1439.2015.05.11 PMC4598521

[29] Liu Q , Ma G , Yang H *et al* Lack of epidermal growth factor receptor gene mutations in exons 19 and 21 in primary lymphoepithelioma‐like carcinoma of the lung. Thorac Cancer 2014; 5: 63–7. 26766974 10.1111/1759-7714.12060 PMC4704286

[30] Chang YL , Yang CY , Lin MW , Wu CT , Yang PC . PD‐L1 is highly expressed in lung lymphoepithelioma‐like carcinoma: A potential rationale for immunotherapy. Lung Cancer 2015; 88: 254–9. 25862146 10.1016/j.lungcan.2015.03.017

[31] Hong S , Liu D , Luo S *et al* The genomic landscape of Epstein‐Barr virus‐associated pulmonary lymphoepithelioma‐like carcinoma. Nat Commun 2019; 10: 3108. 31311932 10.1038/s41467-019-10902-w PMC6635366

[32] Rami‐Porta R , Asamura H , Travis WD , Rusch VW . Lung cancer ‐ major changes in the American Joint Committee on Cancer eighth edition cancer staging manual. CA Cancer J Clin 2017; 67: 138–55. 28140453 10.3322/caac.21390

[33] Zhou Q , Zhang XC , Chen ZH *et al* Relative abundance of EGFR mutations predicts benefit from gefitinib treatment for advanced non‐small‐cell lung cancer. J Clin Oncol 2011; 29: 3316–21. 21788562 10.1200/JCO.2010.33.3757

[34] Kang J , Chen HJ , Zhang XC *et al* Heterogeneous responses and resistant mechanisms to crizotinib in ALK‐positive advanced non‐small cell lung cancer. Thorac Cancer 2018; 9: 1093–103. 29978950 10.1111/1759-7714.12791 PMC6119621

[35] Tam IY , Chung LP , Suen WS *et al* Distinct epidermal growth factor receptor and KRAS mutation patterns in non‐small cell lung cancer patients with different tobacco exposure and clinicopathologic features. Clin Cancer Res 2006; 12: 1647–53. 16533793 10.1158/1078-0432.CCR-05-1981

[36] Wong DW , Leung EL , So KK *et al* The EML4‐ALK fusion gene is involved in various histologic types of lung cancers from nonsmokers with wild‐type EGFR and KRAS. Cancer 2009; 115: 1723–33. 19170230 10.1002/cncr.24181

[37] Fang W , Hong S , Chen N *et al* PD‐L1 is remarkably over‐expressed in EBV‐associated pulmonary lymphoepithelioma‐like carcinoma and related to poor disease‐free survival. Oncotarget 2015; 6: 33019–32. 26361045 10.18632/oncotarget.5028 PMC4741746

[38] Ose N , Kawai T , Ishida D , Kobori Y , Takeuchi Y , Senba H . Pulmonary lymphoepithelioma‐like carcinoma with echinoderm microtubule‐associated protein‐like 4‐anaplastic lymphoma kinase (EML4‐ALK) fusion gene. Respirol Case Rep 2016; 4: e00200. 28031835 10.1002/rcr2.200 PMC5167302

[39] Lin Z , Situ D , Chang X *et al* Surgical treatment for primary pulmonary lymphoepithelioma‐like carcinoma. Interact Cardiovasc Thorac Surg 2016; 23: 41. 26993476 10.1093/icvts/ivw064 PMC4986741

[40] Lin L , Lin T , Zeng B . Primary lymphoepithelioma‐like carcinoma of the lung: An unusual cancer and clinical outcomes of 14 patients. Oncol Lett 2017; 14: 3110–6. 28928848 10.3892/ol.2017.6510 PMC5588446

[41] Zhou C , Xie Z , Qin Y . Genomic profiling of pulmonary lymphoepithelioma‐like carcinoma. ASCO. J Clin Oncol 2019; 37(15 Suppl): Abstract 2019; 1572.

[42] Yin K , Zhi X , Zhang X‐C *et al* PD‐1/PD‐L1 Inhibition Might be an Option for the Treatment of Advanced Primary Pulmonary Lymphoepithelioma‐Like Carcinoma. World Conference on Lung Cancer P204‐30. J Thorac Oncol 2018; 13 (10 Suppl): S742.

[43] Wu YL , Zhong WZ , Li LY *et al* Epidermal growth factor receptor mutations and their correlation with gefitinib therapy in patients with non‐small cell lung cancer: A meta‐analysis based on updated individual patient data from six medical centers in mainland China. J Thorac Oncol 2007; 2: 430–9. 17473659 10.1097/01.JTO.0000268677.87496.4c

[44] An SJ , Chen ZH , Su J *et al* Identification of enriched driver gene alterations in subgroups of non‐small cell lung cancer patients based on histology and smoking status. PLOS One 2012; 7: e40109. 22768234 10.1371/journal.pone.0040109 PMC3387024

[45] Canale M , Petracci E , Delmonte A *et al* Impact of TP53 mutations on outcome in EGFR‐mutated patients treated with first‐line tyrosine kinase inhibitors. Clin Cancer Res 2017; 23: 2195–202. 27780855 10.1158/1078-0432.CCR-16-0966

[46] Anand A , Zayac A , Curtiss C , Graziano S . Pulmonary lymphoepithelioma‐like carcinoma disguised as squamous cell carcinoma. J Thorac Oncol 2018; 13: e75–6. 29703540 10.1016/j.jtho.2017.11.133

